# Supplementary material for: Herpes Simplex Virus 1 Infection of Human Brain Organoids and Pancreatic Stem Cell-Islets Drives Organoid-Specific Transcripts Associated with Alzheimer’s Disease and Autoimmune Diseases
Source: Cells. 2024 Nov 29;13(23):1978. doi: 10.3390/cells13231978 (PMC11640215; doi:10.3390/cells13231978)
Supplement: Supplementary file 1 [file cells-13-01978-s001.zip › cells-3128959_supp.pdf]

Supplementary Figures

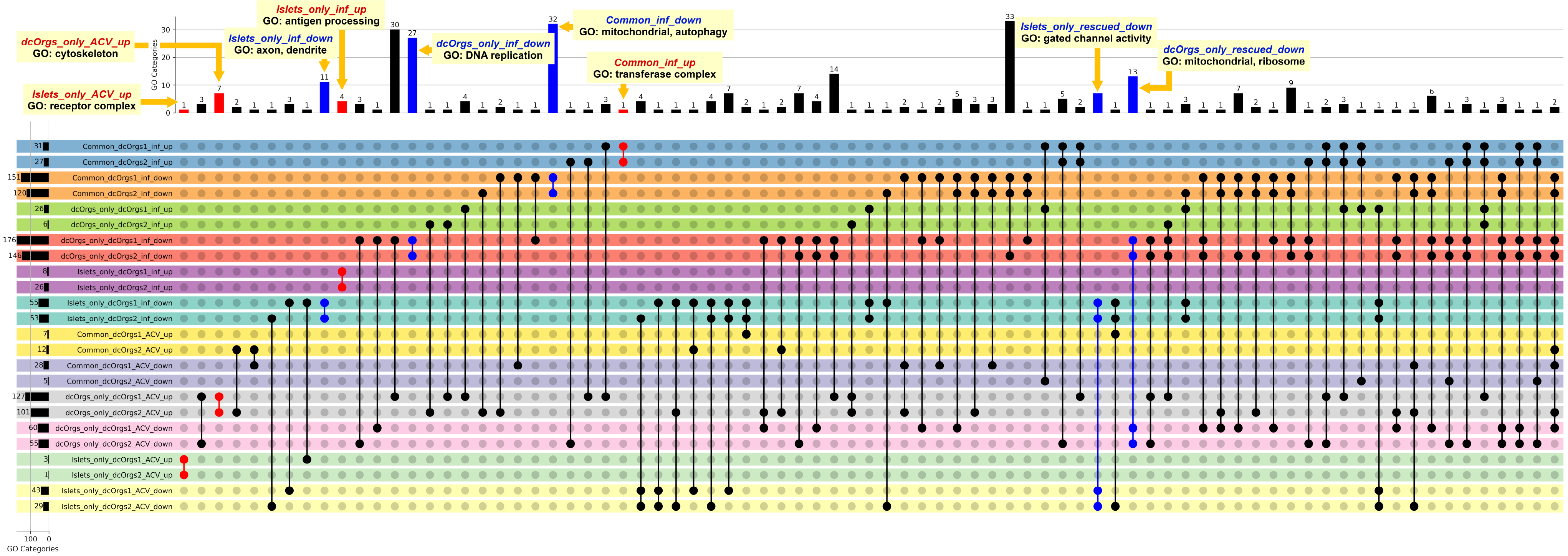

Figure S1: Full set of shared GO categories across HSV-1-infected, or HSV-1-infected and ACV-treated sc-islets and dcOrgs.

The bar graph at the top shows the numbers of shared GO categories across the DEG results, and the UpSet plot shows the DEG results that share these distinct GO categories. Shaded circles indicate the DEG result that share the GO category; the red shaded circles highlight the up-regulated DEG results with GO categories that were mentioned in the main text; the blue shaded circles highlight the down-regulated DEG results with GO categories that were mentioned in the main text. The first four rows in the UpSet plot show the shared GO categories for the up-regulated genes in common due to HSV-1 infection in sc-islets and both sets of dcOrg replicates (Common_dcOrgs1_inf_up and Common_dcOrgs2_inf_up), down-regulated genes in common in HSV-1-infected sc-islets and both dcOrg replicates (Common_dcOrgs1_inf_down and Common_dcOrgs2_inf_down). The next eight rows in the UpSet plot show the shared GO categories for up-regulated or down-regulated genes exclusively in dcOrgs only, or exclusively in islets only. The results for the HSV-1-infected and ACV-treated datasets are shown in the next 12 rows.
